# Modelling the significance of website quality and online reviews to predict the intention and usage of online hotel booking platforms

**DOI:** 10.1016/j.heliyon.2022.e10735

**Published:** 2022-09-23

**Authors:** Anas A. Salameh, Abdullah Al Mamun, Naeem Hayat, Mohd Helmi Ali

**Affiliations:** a College of Business Administration, Prince Sattam Bin Abdulaziz University, Al-Kharj 11942, Saudi Arabia; b UKM-Graduate School of Business, Universiti Kebangsaan Malaysia, 43600, UKM Bangi, Selangor Darul Ehsan, Malaysia; c Global Entrepreneurship Research and Innovation Centre, Universiti Malaysia Kelantan, Pengkalan Chepa, 16100 Kota Bharu, Kelantan, Malaysia

**Keywords:** Online hotel booking, Reviews, Usefulness, Service quality, System quality, Malaysia

## Abstract

Innovative technologies are paving the way for the online purchase of products and services. The COVID-19 also harness online shopping and hotel booking in the post-COVID-19 era. Online hotel booking is becoming the norm among young consumers. The six determinants of the intention to use online hotel booking platforms (OHBPs) are information quality, integrity, perceived risks, perceived benefits, system quality, and service quality was utilized in the study. Cross-sectional data were collected through the online survey, and 884 valid responses were utilised for the data analysis. The analysis results showed that the perceived benefits, system quality, and service quality significantly predicted the intention to use the OHBPs. Meanwhile, the usefulness and quantity of online reviews and the intention to use OHBPs have a positive and significant effect on the usage of the OHBPs. The study results confirmed the insignificant moderating role of the usefulness and quantity of online hotel booking reviews in the relationship between the intention to use and the usage of OHBPs. The intention to use OHBPs had low predictive power, while the usage of OHBPs had high predictive power. This study’s findings have offered valuable theoretical and managerial contributions. The management of OHBPs needs to concentrate on information quality and integrity to help manage the associated risks of online hotel booking. Previous customers' reviews are vital to encourage the usage of OHBPs. Finally, social media usage and promoting the OHBPs by sharing satisfied users' experiences improve the intention and usage of the OHBPs.

## 1 Introduction

Information and communication technologies (ICT) bring multiple social and technological changes to the global population (Chi, 2018). The rise of internet technologies and Web 2.0 applications promotes social media and web-based applications, creating various types of virtual communities and networks (Jou and Day, 2021). The tourism industry has heavily adopted ICT to promote and facilitate hospitality services (Hayat and Al-Mamun, 2020). Online hotel booking is only one aspect of technology usage in tourism.

The use of ICT-based online shopping platforms and mobile-based applications for travel, car, and air ticket booking is on the rise around the globe (Chi, 2018). Consumers' purchases significantly rely on the right and complete information from the product or service providers (Zhao et al., 2015). Nonetheless, websites pose challenges that restrict online consumers' purchase decisions. Lack of complete information and trust are the few significant factors that reduce consumers' confidence in making purchases online (Ponte et al., 2015). Online purchasing offers the benefits of flexibility and ease of use. The websites offering services and products are a system and need to have system-level quality and service-level quality, promoting positive inclination among the users (Tien et al., 2019). The use of technology has increased in the hotel and hospitality industry, looking to improve customer experience in online hotel booking (Tao et al., 2018). Online hotel booking is like the purchase of services online. Customers are looking for complete and trustworthy information highlighting the services' benefits with the right system level and service quality (Kim et al., 2017).

On the other hand, the online shopper opinions and reviews about the online booking service quality help increase the usage of online hotel booking platforms (OHBPs) (Park and Huang, 2017). Web 2.0 technologies assist online users in posting their service experiences, both bad and good (Tussyyadiah and Park, 2018). The online shoppers show concern about the non-fulfilment of the promises made by the online sellers and offer reviews about the online services (Zhao et al., 2015). Online reviews must be helpful and in sufficient numbers to help prospective online customers make up their minds. The usefulness of online reviews helps prospective online users in the purchase decision process (Tien et al., 2019). Satisfied online users narrate their experience and create effective suggestions for other users to confidently use the online services (Tussyadiah and Park, 2018). Satisfied online users facilitate prospective users to think positively about online services or websites. Online user reviews act like electronic word of mouth (eWOM) (Park and Lee, 2009).

As an emerging economy, Malaysia relies heavily on tourism, attracting more than 25 million international tourists annually prior to COVID-19 (Tourism Malaysia, 2020), and is regarded as a low-cost travel destination. The tourism sector's contribution to the GDP reached 12% and provided 15% of employment in the country. Most tourists are inclined to use web resources to book hotels, and intensive use of social media is evident in recent times (Hayat and Al-Mamun, 2020). Social media empowers tour operators, hotel management, and prospective customers to understand the consumer's needs and deliver the best services (Jou and Day, 2021). Online hotel booking requires a manageable and state-forward web platform to book the hotel online. The quality attributes of the online platform facilitate users' ability to gain information and quickly make hotel bookings (Park and Huang, 2017). Young users are also attracted to posting hotel reviews on social media and the hotel website.

Online hotel booking, like online shopping, requires the appropriate information on the right technology platform to build the intention to use the online platform (Chi, 2018). Information integrity and the right product and service information benefits prospective users (Jou and Day, 2021). The users are always looking for the website quality and quality of hotel services. Thus, the current study investigates the formation of users' intention to use and usage of OHBPs among Malaysian respondents. However, the prospective tourist like to see and use online reviews to evaluate the hotel's quality. The usability of the review and the volume of online reviews also play a vital role in forming the user's behaviour towards using the online book platforms (Tussyadiah and Park, 2018). In the case of OHBPs, the online hotel booking users must reliably attain complete hotel information. The associated service and system-level quality of the OHBPs help build the positive motivation to use the OHBPs. Furthermore, the existing online hotel booking user reviews in terms of usefulness and quantity help to nurture the usage of the OHBPs.

## 2 Literature review

### 2.1 Theoretical foundation

Most of the recent works have proposed using the technology adoption model (TAM) to explore the adoption of OHBPs. Nevertheless, limited empirical works have suggested exploring the intention to adopt and the usage of OHBPs with the website quality attributes (Jou and Day, 2021). Website quality is a multidimensional construct influencing the consumers' attitudes and causing satisfaction (Anas et al., 2018). Online users need information stimuli to attract them towards online shopping platforms. Chi (2018) has asserted that information quality and trust in service promote online users' positive attitudes towards online shopping platforms. The use of technology offers unique experiences to the consumers, and users look for benefits and a low level of risk while using the technologies.

Online shopping platforms are systems and require the necessary system-level quality and service delivery (Chi, 2018). Response time, security, and well-designed websites offer a rich user experience to help satisfy and prompt the intention to use online shopping and booking platforms. Due to the rise in social media and Web 2.0, users' online reviews have gained importance for prospective technology users or online shoppers (Duan et al., 2008). Online hotel booking reviews act as WOM and facilitate prospective users to shop online. Two aspects of online reviews are essential and influence prospective online customers: the usefulness and quantity of the online reviews as perceived by prospective online users (Zhao et al., 2015). The proposed model in this study is based on the website quality attributes leading to the formation of the intention to use OHBPs, online reviews' usefulness and quantity, and the intention to use OHBPs to promote online hotel booking.

### 2.2 Hypotheses development

#### 2.2.1 Formation of intention to use OHBPs

Decision-makers look for the right information at the right time in the most suitable manner. The information provided to prospective users on online platforms must be comprehensive and relevant in order for them to make informed decisions about the products and services (Yang, 2012). Information quality is crucial for activities utilising web technologies such as online shopping, ticket booking, and hotel booking (Tran et al., 2017). The online information must be clear, concise, adequate, and valuable to web users. Chi (2018) postulated that the website information quality reflects the intention to use the online web platform for e-commerce. For the current study, we would like to propose the following:

H1 *Information quality positively affects the intention to use OHBPs.*

Using ICT brings multiple benefits to users, such as time-saving, cost reduction, and convenience in online shopping (Kim et al., 2019). For example, OHBPs provide hotel options based on the preferences of customers and within their budget (Agag et al., 2020). The web platforms provide extensive and accurate hotel room information, pricing, and availability, enticing potential customers to book (Anas et al., 2018). The following hypothesis is proposed:

H2 *Integrity positively affects the intention to use OHBPs.*

The information available on the website also builds the perception of benefits. Online shopping also offers the benefits of personalization, with ubiquitous and enabling platforms to explore the product and service options in detail (Hahn et al., 2017). These prospective customers can find a hotel in a specific location according to their preferred time and price range (Park and Huang, 2017). Thus, the perceived benefits of online hotel booking influence the intention to use the OHBPs (Tran et al., 2017). Therefore, we forward the next hypothesis:

H3 *Perceived benefits positively affect the intention to use OHBPs.*

On the other hand, the perception of risk emerges among the consumers regarding unwanted situations and negative consequences associated with online purchasing (Gurang et al., 2016). For instance, online shopping is highly risky as the users' personal and financial information must be entered to complete the online transaction (Ponte et al., 2015). The higher potential for risks negatively influences the intention of making a purchase online. Tao et al. (2018) postulated that hotel booking via OHBPs requires time and disclosure of sensitive personal information, negatively influencing consumers' willingness to use the OHBPs. We offer the following hypothesis:

H4 *Perceived risks negatively affect the intention to use OHBPs.*

Websites are systems with multiple attributes presented to the users for them to promptly perform the required task (Anas et al., 2018). Website connectivity and swift response from the website denote a web portal's system quality (Agag et al., 2020). For hotel booking platforms, the error-free response and ease of use of the web platform represent the system quality of the hotel booking platform (Elwalda et al., 2016). OHBPs' system quality instigates the intention to use the online booking platform. Here the next hypothesis is suggested:

H5 *System quality positively affects the intention to use OHBPs.*

Service quality represents the service platform’s overall customer evaluation of service quality (Wang and Wang, 2010). The perception of service quality instigates consumer behaviour (Hahn et al., 2017). E-service quality has become an essential area of concern for business enterprises (Agag et al., 2020). Most business activities are performed online, such as online shopping for fashion and electronic ticket booking for trains and flights (Ozturk et al., 2017). Facilitation and ease can reduce the consumer's effort and harness the intention to use online shopping platforms (Agag et al., 2020). The following hypotheses are proposed in this study:

H6 *Service quality positively affects the intention to use OHBPs.*

### 2.3 Usage of OHBPs

Online reviews posted by past customers help new customers better understand the product or services (Duan et al., 2008). A customer visits the OHBPs and utilizes the information posted by prior customers via Web 2.0, which helps the customer to book a hotel room online (Papathanassis and Knolle, 2011; Zhu et al., 2016). The usefulness of online reviews is that they aid in determining the customers' intention to use and usage of OHBPs (Ozturk et al., 2016). The relevancy and reliability of the reviews suggest that the information is valuable in helping new customers book a hotel room online (Park and Lee, 2009). The online reviews will be helpful when they are genuine and offer complete information about the hotel, encouraging the customers to book via an online platform (Tussyadiah and Park, 2018).

Besides, the volume of online reviews is a significant feature of eWOM (Park and Lee, 2009). The number of positive and negative comments attracts the information seeker and offers significant awareness of the product and services (Zhao et al., 2015). The online reviews also reflect the usage of OHBPs and show the hotels' booking acceptance.

H7 *The usefulness of online reviews positively affects the usage of the OHBPs.*

H8 *The volume of online reviews positively affects the usage of the OHBPs*

Elwalda et al. (2016) have postulated that online booking reviews increase insignificantly predict online booking sales. Nonetheless, online reviews suggest the users' higher acceptance and reduce the perceived risk of booking hotel rooms online (Papathanassis and Knolle, 2011). Hence, the following hypotheses are proposed:

H9 *Intention to use OHBPs positively affects the usage of the OHBPs.*

### 2.4 Moderation by usefulness and volume of online reviews

Positive online reviews increase the usage of OHBPs (Elwada et al., 2016). Consumers have varied perceptions of online reviews, and many consumers perceive online reviews as valuable, while numerous others think otherwise (Tien et al., 2019). Regardless, the intention to use online platforms positively influences online platforms' usage. Therefore, the usefulness of the online reviews may moderate the relationship between the intention to use and the usage of OHBPs (Zhu et al., 2016). This study suggests that the perception of the usefulness of online reviews for hotel booking moderates the relationship between the intention to use OHBPs and the usage of OHBPs. Similarly, this study proposes that the quantity or volume of online reviews for hotel booking moderates the relationship between the intention to use and usage of OHBPs. The hypotheses are as follows:

HM1: *The relationship between the intention to use OHBPs and the usage of OHBPs is moderated by the usefulness of online reviews.*

HM2: *The relationship between the intention to use OHBPs and the usage of OHBPs is moderated by the volume of online reviews.*

## 3 Research methodology

### 3.1 Research design

A survey-based quantitative method was employed in this study to explore the factors impacting the intention to use and the usage of OHBPs. Data were collected in a cross-sectional manner for this explanatory research. The causal-predictive data analysis technique, partial least squares structural equation modelling (PLS-SEM), was utilised for hypothesis testing.

### 3.2 Population and sample

The target population of the current study was potential and current users of OHBPs. The sample size calculation was performed with G-Power 3.1 with a power of 0.95 and an effect size of 0.15 with seven predictors. The required sample size was 145 (Faul et al., 2007). A minimum threshold of 200 samples is suggested for PLS-SEM (Hair et al., 2019). This study intended to employ the second-generation statistical analysis technique of structural equation modelling; therefore, data were collected from more than 800 respondents. The convenience sampling technique was utilized, and a few qualifying questions were added to the survey by taking the respondents' consent to participate in the study. Data collection was performed using a google form posted on social media platforms, including Facebook and WhatsApp, from July 2020 to August 2020.

### 3.3 Survey instrument

The current study utilized pretested and validated scales. Five items taken from Wang and Wang (2010) evaluated the hotel booking information quality, while OHBPs' integrity was assessed using five items adapted from Agag et al. (2020). Next, the perceived benefits of the OHBPs were estimated with the five items taken from Park and Huang (2017). In contrast, website attributes of perceived risk, system quality, and website service quality were assessed with five items improvised from the work of Wang and Wang (2010). Intention to use the OHBPs was assessed with five items from Park and Huang’s work (2017) and Agag et al. (2020).

Meanwhile, the usefulness and volume of online reviews were evaluated with five items, each taken from the work of Zhao et al. (2015). Finally, usage of OHBPs was evaluated with a single-question item. The survey instrument used in this study was a structured questionnaire, and all the question items were adopted from earlier studies with minor modifications. In this study, a seven-point Likert scale (strongly disagree, disagree, somewhat disagree, neither agree nor disagree, somewhat agree, agree, and strongly agree) was used to measure the intention to use and usage of OHBPs. All independent and moderating variables were measured using a five-point Likert scale (strongly disagree, disagree, neither agree nor disagree, agree and strongly agree).

### 3.4 Common method bias (CMB)

Cross-sectional studies are commonly associated with common method bias; CMB is assessed using multiple methodological and statistical tools (Podsakoff et al., 2012). Therefore, the current study applied Harman’s one-factor test to determine CMV’s effect as a diagnostic technique. The single factor accounted for 36.8%, which was below the recommended threshold of 40% in Harman’s one-factor test, thus, approving the inconsequential influence of CMV on this study (Podsakoff et al., 2012). The study also evaluated the CMV following Kock’s (2015) recommendation to test the full collinearity of all the constructs. All the study constructs regressed on the common variable, and the variance inflation factor (VIF) values were 2.905 for information quality, 2.692 for the integrity of OHBPs, 2.181 for perceived benefits, 1.191 for perceived risk, 3.093 for system quality, 2.572 for service quality, 2.338 for intention to use OHBPs, 2.820 for the usefulness of online reviews, 2.666 for the volume of online reviews, and 1.677 for the usage of OHBPs. All the VIF values were less than 3.3, indicating the absence of bias from the single-source data.

### 3.5 Multivariate normality

Hair et al. (2019) suggested evaluating multivariate normality data before using the SmartPLS. Therefore, multivariate normality for the study data was assessed with the Web Power online tool (source: https://webpower.psychstat.org/wiki/tools/index). The calculated Mardia’s multivariate p-value revealed that the study data had a non-normality issue as the *p*-values were below 0.05 (Cain et al., 2017).

### 3.6 Data analysis method

Due to the existence of multivariate non-normality in the dataset, PLS-SEM was utilised. Hair et al. (2014) have recommended that variance-based structural equation modelling be adopted to analyse this exploratory nature and non-normality issue to explain variance in the structural equation model’s dependent constructs in-depth.

The Smart-PLS 3.1 program was employed to analyse the data collected in the current study. PLS-SEM is a multivariate exploratory method for analysing integrated latent constructs' path structure (Hair et al., 2019). It empowers researchers to work well with non-normal data in a small dataset. Furthermore, PLS-SEM is a casual-predictive analytical tool that executes complex models with composites and has no specific assumption of the goodness-of-fit static requirements (Hair et al., 2014). The PLS-SEM analysis was performed in two phases. The first step dealt with model estimation, where the constructs' reliability and validity were evaluated (Hair et al., 2019). Phase two dealt with evaluating the models' correlations and the path model's systematic testing (Hair et al., 2014). Analysis was performed with *r*^*2*^, Q^2^, and effect size f^2^ explaining the endogenous construct’s change caused by the exogenous constructs (Hair et al., 2019).

The importance-performance map analysis (IPMA) categorizes the study constructs into relatively high to low by their corresponding importance and the performance of the endogenous construct (Hair et al., 2014). IMPA differentiates the possible area of improvement from the practice and scholarly standpoints. The IPMA analysis alters the total effect into rescaled variables totals as an unstandardized method (Ringle and Sarstedt, 2016). All the latent constructs' ranges are rescaled from 0 to 100. The latent construct’s mean score signifies the performance of the latent construct, where 0 indicates the smallest, while 100 indicates the highest importance in the performance of the endogenous construct (Hair et al., 2019).

## 4 Data analysis

### 4.1 Demographic features of respondents

The demographic analysis, as presented in Table 1, revealed that 53.8% of the study respondents were men. Moreover, 90.6% of the study respondents were single, while 8.3% were married, and the remaining were divorced or windowed. Most of the study respondents were between 21 and 30 with 41.4%, whereas the others were below 21 years old (40.4%), 31–40 years old (11.4%), and the rest were more than more than 40 years old. Among the 884 respondents, 58.5% had a bachelor's level education, 18.7% had a secondary school level education, 19.0% had a diploma level education, and the remaining had a higher than college degree-level education. Furthermore, 70.7% of respondents had a monthly income of less than RM 2500, 17.9% had between RM 2501 and RM 5000, 7.5% had between RM 5001 and RM 10,000, while the remaining respondents had a monthly income of more than RM 10,000. Most of the respondents were Chinese, and the remaining 75.6% comprised respondents of other ethnicities. Finally, most of the respondents resided in urban areas.

**Table 1:** Demographic characteristics of respondents.

	N	%		N	%
*Gender*			*Marital Status*		
Female	476	46.2	Single	801	90.6
Male	408	53.8	Married	73	8.3
Total	884	100.0	Divorced	8	0.9
			Widowed	2	0.2
*Age Group*			Total	884	100.0
Below 21 years	360	40.7			
21–30 years	366	41.5	*Education*		
31–40 years	101	11.4	Secondary school certificate	165	18.7
41–50 years	33	3.7	Diploma certificate	168	19.0
More than 51 years	24	2.7	Bachelor degree or equivalent	517	58.5
Total	884	100.0	Master's degree	27	3.1
			Doctoral degree	7	0.8
*Average Monthly Income* (RM)			Total	884	100.0
Below 2500	625	70.7			
2501 to 5000	159	17.9	*Ethnicity*		
5001 to 10,000	67	7.5	Malay	68	7.7
More than 10,000	33	3.7	Chinese	668	75.6
Total	884	100.0	Indian	59	6.7
			Others	89	10.1
*Residential Area*			Total	884	100.0
Urban	775	87.7			
Rural	109	12.3			
Total	884	100.0			

### 4.2 Reliability and validity

Hair et al. (2019) recommended a two-step data evaluation followed in this study. In stage one, the study’s latent constructs' reliabilities and validities were attained and assessed using Cronbach’s alpha (CA), Dijkstra-Hensele’s *rho (*rho_A), and composite reliability (CR). Cronbach’s alpha scores for every study construct were more than the 0.7 thresholds, and the minimum value of Cronbach’s alpha was 0.771 (Hair et al., 2014). The results are depicted in Table 2. Additionally all the Dijkstra-Hensele's *rho* scores for the study constructs were more than the 0.7 threshold, where the minimum value of Dijkstra-Hensele’s *rho* was 0.790 (Hair et al., 2019).

**Table 2:** Reliability and validity results.

Variables	No. Items	Mean	SD	CA	rho_A	CR	AVE	VIF
INQ	5	3.807	0.711	0.831	0.833	0.881	0.596	2.885
IGH	5	3.863	0.671	0.863	0.867	0.902	0.648	2.583
PRB	5	4.113	0.613	0.809	0.812	0.867	0.566	2.085
PRK	5	3.602	0.803	0.848	0.881	0.888	0.614	1.169
SQL	5	4.015	0.614	0.771	0.790	0.845	0.524	2.550
SRL	5	3.927	0.646	0.814	0.818	0.870	0.574	2.428
IHB	5	5.673	1.022	0.912	0.912	0.934	0.740	2.212
UOR	5	5.437	0.976	0.875	0.880	0.909	0.666	2.490
VOR	5	5.646	0.990	0.879	0.887	0.912	0.674	2.576
UHB	1	3.690	1.144	1	1	1	1	-

*Note:* INQ: Information Quality; IGH: Integrity of online hotel booking; PRB: Perceived benefits; PRK: Perceived risk; SQL: System quality, SRL: Service quality; IHB: Intention to use OHBPs; UOR: Usefulness of online reviews; VOR: Volume of online reviews; UHB: Usage of OHBPs. SD: Standard Deviation; CA: Cronbach's Alpha; *rho_A -* Dijkstra-Hensele's *rho*; CR - Composite Reliability; AVE - Average Variance Extracted; VIF - Variance Inflation Factors.

Similarly, the CR scores were over the 0.7 threshold, where the lowest CR value was 0.867 (Hair et al., 2014). These results indicated that the latent constructs achieved appropriate reliabilities and performed well in the next stage of the analysis. The average value extracted (AVE) for each construct must be over 0.50 to have acceptable convergent validity to achieve each construct’s uni-dimensionality (Hair et al., 2019). Each construct in this study had adequate convergent validity (see Table 3). Meanwhile, the VIF value for each construct was below the threshold of 3.3, confirming the no issue of multicollinearity (Hair et al., 2014). The item loading and cross-loading were reported to confirm the constructs' discriminant validity, as listed in Tables 3 and 4.

**Table 3:** Discriminant validity results.

	INQ	IHB	PRB	PRK	SQL	SRL	IHB	UOR	VOR	UHB
*Fornell-Larcker Criterion*										
INQ	0.772									
IHB	0.733	0.805								
PRB	0.612	0.631	0.752							
PRK	0.283	0.288	0.268	0.784						
SQL	0.672	0.647	0.584	0.373	0.757					
SRL	0.696	0.615	0.650	0.280	0.677	0.724				
IHB	0.524	0.475	0.550	0.191	0.544	0.644	0.860			
UOR	0.570	0.547	0.546	0.303	0.601	0.621	0.678	0.816		
VOR	0.487	0.433	0.543	0.278	0.549	0.633	0.678	0.733	0.821	
UHB	0.312	0.327	0.434	0.023	0.312	0.442	0.591	0.480	0.486	1.000
*HTMT ratios*										
INQ	-									
IHB	0.867	-								
PRB	0.745	0.757	-							
PRK	0.335	0.332	0.311	-						
SQL	0.817	0.774	0.721	0.446	-					
SRL	0.883	0.776	0.822	0.349	0.876	-				
IHB	0.593	0.533	0.637	0.202	0.627	0.756	-			
UOR	0.670	0.634	0.649	0.349	0.713	0.761	0.751	-		
VOR	0.566	0.494	0.639	0.311	0.645	0.759	0.756	0.834	-	
UHB	0.336	0.351	0.479	0.037	0.344	0.492	0.618	0.508	0.515	-

*Note:* INQ: Information Quality; IGH: Integrity of online hotel booking; PRB: Perceived benefits; PRK: Perceived risk; SQL: System quality, SRL: Service quality; IHB: Intention to use OHBPs; UOR: Usefulness of online reviews; VOR: Volume of online reviews; UHB: Usage of OHBPs.

**Table 4:** Loadings and cross-loading results.

Code	INQ	IGH	PRB	PRK	SQL	SRL	IHB	UOR	VOR	UHB
INQ1	*0.784*	0.594	0.443	0.202	0.478	0.492	0.352	0.411	0.311	0.186
INQ2	*0.802*	0.586	0.483	0.200	0.513	0.536	0.403	0.439	0.364	0.227
INQ3	*0.747*	0.525	0.481	0.239	0.556	0.520	0.364	0.459	0.389	0.229
INQ4	*0.804*	0.630	0.497	0.232	0.555	0.561	0.400	0.460	0.392	0.242
INQ5	*0.720*	0.498	0.453	0.217	0.565	0.482	0.473	0.427	0.405	0.300
IGH1	0.554	*0.808*	0.534	0.208	0.522	0.504	0.406	0.451	0.386	0.282
IGH2	0.599	*0.821*	0.503	0.227	0.458	0.499	0.335	0.418	0.308	0.249
IGH3	0.611	*0.836*	0.505	0.227	0.511	0.532	0.398	0.455	0.360	0.291
IGH4	0.609	*0.821*	0.514	0.246	0.513	0.569	0.406	0.462	0.366	0.256
IGH5	0.579	*0.733*	0.480	0.254	0.461	0.492	0.356	0.408	0.312	0.234
PRB1	0.405	0.410	*0.766*	0.217	0.459	0.419	0.395	0.390	0.373	0.303
PRB2	0.459	0.443	*0.774*	0.164	0.503	0.424	0.454	0.395	0.437	0.350
PRB3	0.444	0.437	*0.762*	0.211	0.502	0.475	0.422	0.429	0.435	0.359
PRB4	0.489	0.534	*0.721*	0.233	0.456	0.429	0.356	0.397	0.343	0.261
PRB5	0.508	0.559	*0.737*	0.192	0.520	0.450	0.432	0.443	0.441	0.348
PRK1	0.177	0.209	0.228	*0.791*	0.248	0.306	0.178	0.247	0.280	0.041
PRK2	0.280	0.271	0.228	*0.785*	0.231	0.315	0.141	0.251	0.179	0.002
PRK3	0.243	0.244	0.242	*0.834*	0.253	0.300	0.191	0.254	0.234	0.043
PRK4	0.165	0.150	0.138	*0.746*	0.153	0.241	0.091	0.185	0.168	0.006
PRK5	0.247	0.241	0.170	*0.761*	0.163	0.289	0.096	0.233	0.189	-0.041
SQL1	0.555	0.468	0.488	0.246	*0.743*	0.520	0.449	0.476	0.443	0.318
SQL2	0.512	0.437	0.466	0.210	*0.764*	0.498	0.466	0.458	0.449	0.312
SQL3	0.415	0.365	0.453	0.088	*0.733*	0.405	0.499	0.394	0.498	0.359
SQL4	0.526	0.456	0.535	0.224	*0.792*	0.514	0.559	0.493	0.539	0.384
SQL5	0.560	0.570	0.407	0.292	*0.566*	0.566	0.317	0.446	0.329	0.187
SRL1	0.529	0.537	0.461	0.334	0.532	*0.716*	0.350	0.432	0.362	0.215
SRL2	0.556	0.504	0.464	0.277	0.534	*0.768*	0.428	0.461	0.428	0.221
SRL3	0.501	0.496	0.457	0.241	0.521	*0.778*	0.426	0.487	0.424	0.247
SRL4	0.462	0.465	0.410	0.277	0.477	*0.761*	0.388	0.410	0.414	0.228
SRL5	0.502	0.459	0.425	0.295	0.504	*0.761*	0.456	0.481	0.442	0.265
IHB1	0.463	0.429	0.495	0.202	0.527	0.474	*0.837*	0.552	0.534	0.486
IHB2	0.448	0.385	0.479	0.139	0.556	0.473	*0.882*	0.565	0.561	0.505
IHB3	0.428	0.412	0.445	0.165	0.549	0.457	*0.873*	0.587	0.589	0.527
IHB4	0.466	0.428	0.493	0.168	0.574	0.473	*0.863*	0.616	0.612	0.532
IHB5	0.446	0.390	0.453	0.150	0.564	0.464	*0.844*	0.592	0.619	0.490
UOR1	0.429	0.422	0.440	0.219	0.520	0.446	0.621	*0.820*	0.591	0.442
UOR2	0.519	0.509	0.489	0.282	0.514	0.523	0.554	*0.844*	0.612	0.378
UOR3	0.506	0.499	0.451	0.263	0.479	0.503	0.538	*0.845*	0.565	0.353
UOR4	0.447	0.468	0.419	0.295	0.473	0.497	0.454	*0.768*	0.550	0.340
UOR5	0.435	0.354	0.430	0.193	0.534	0.493	0.572	*0.800*	0.659	0.426
VOR1	0.385	0.323	0.427	0.227	0.510	0.446	0.551	0.611	*0.814*	0.383
VOR2	0.423	0.391	0.466	0.258	0.557	0.476	0.574	0.646	*0.844*	0.410
VOR3	0.404	0.377	0.458	0.220	0.531	0.474	0.596	0.602	*0.854*	0.444
VOR4	0.396	0.352	0.472	0.201	0.534	0.459	0.577	0.596	*0.854*	0.421
VOR5	0.396	0.333	0.404	0.242	0.464	0.392	0.479	0.559	*0.733*	0.324
UOB	0.312	0.327	0.434	0.023	0.442	0.312	0.591	0.480	0.486	*1*

*Note:* INQ: Information Quality; IGH: Integrity of online hotel booking; PRB: Perceived benefits; PRK: Perceived risk; SQL: System quality, SRL: Service quality; IHB: Intention to use OHBPs; UOR: Usefulness of online reviews; VOR: Volume of online reviews; UHB: Usage of OHBPs. The Italic values in the matrix above are the item loadings, and others are cross-loadings.

The constructs achieved apposite discriminant validities (see Table 3). Besides, the Fornell-Larcker criterion (1981) and the HTMT ratio measure the constructs' discriminant validity. The Fornell-Larcker criterion was assessed with the square root of the individual construct’s AVE, which must be higher than the correlation among the other constructs (Hair et al., 2019). On the other hand, the HTMT ratio required less than 0.90 to establish discriminant validity for each construct (Henseler et al., 2015). Tables 3 and 4 show that each construct has discriminant validity in this study.

### 4.3 Path analysis

After accomplishing the adequate reliabilities and validities from the study model’s structural evaluation, measurement assessment was employed to test the study’s hypotheses. The adjusted *r*^*2*^ score for the input constructs of information quality (INQ), the integrity of online hotel booking (IGH), perceived benefits (PRB), perceived risk (PRK), system quality (SQL), and service quality (SRL) on the intention to use OHBPs (IHB) explained 45.5% of the change in the intention to use the OHBPs. The predictive relevance (Q^2^) score for that part of the model was 0.334, representing a considerable predictive relevance (Hair et al., 2014). Next, the adjusted *r*^*2*^ score for the three exogenous constructs, i.e., the usefulness of online reviews (UOR), volume of online reviews (VOR), and (IHB) on the Usage of OHBPs (UHB) clarified 36.4% of variations in the usage of OHBPs. The predictive relevance (Q^2^) score for that part of the model was 0.357, indicating a medium predictive relevance (Hair et al., 2014).

The model’s standardised path scores, t-values, and significance level are shown in Table 5. The path coefficient for the INQ and IHB (β = 0.042, t = 0.862, *p* = 0.195) denoted a positive and insignificant effect. This suggests that the information quality does not influence the intention to use OHBPs, thus rejecting H_1_. The path score between IGH and IHB (β = –0.014, t = 0.294, *p* = 0.384) indicated an insignificant and negative influence of the integrity of OHBPs on the intention to use OHBPs. This outcome formed insignificant statistical support to accept H_2_. Next, the path coefficient for the PRB and IHB (β = 0.189, t = 4.091, *p* = 0.000) symbolized the positive and significant effect of perceived benefits on the intention to use OHBPs. This suggests support for H_3_.

**Table 5:** Path coefficients results.

Hypo		Beta	*T*	*P*	*r*^2^	*f*^*2*^	Q^2^	Decision
H_1_	INQ → IHB	0.042	0.862	0.195		0.001		Reject
H_2_	IGH → IHB	-0.014	0.294	0.384		0.000		Reject
H_3_	PRB → IHB	0.189	4.091	0.000	0.458	0.032	0.334	Accept
H_4_	PRK → IHB	-0.039	1.331	0.092		0.002		Reject
H_5_	SQL → IHB	0.408	8.161	0.000		0.121		Accept
H_6_	SRL → IHB	0.153	3.603	0.000		0.018		Accept
H_7_	UOR → UHB	0.094	2.126	0.017		0.006		Accept
H_8_	VOR → UHB	0.110	2.513	0.006	0.367	0.007	0.357	Accept
H_9_	IHB → UHB	0.443	10.061	0.000		0.140		Accept

*Note:* INQ: Information Quality; IGH: Integrity of online hotel booking; PRB: Perceived benefits; PRK: Perceived risk; SQL: System quality, SRL: Service quality; IHB: Intention to use OHBPs; UOR: Usefulness of online reviews; VOR: Volume of online reviews; UHB: Usage of OHBPs.

Meanwhile, the path from PRK to IHB (β = –0.039, t = 1.331, *p* = 0.092), which explained the perceived risk's impact on the intention to use OHBPs, was negative and significant. It did not support the acceptance of H_4_. The path value for the SQL and IHB (β = 0.408, t = 8.161, *p* = 0.000) showed that the system quality impacted the intention to use OHBPs. Hence, the result supports the acceptance of H_5_. Besides, the path between SRL and IHB (β = 0.153, t = 3.603, *p* = 0.000), indicating the influence of the service quality on the intention to use OHBPs, was positive and significant. It supports the acceptance of H_6_. The path between UOR and UHB (β = 0.094, t = 2.126, *p* = 0.017), demonstrating the influence of online reviews on the usage of OHBPs, was positive and significant, thus, supporting H_7_. Next, the path between VOR and UHB (β = 0.110, t = 2.513, *p* = 0.006), representing the influence of the volume of online reviews on the usage of OHBPs, was positive and significant. It supports the acceptance of H_8_. Finally, the path coefficient for the IHB and UHB (β = 0.443, t = 10.061, *p* = 0.000) signified a positive and significant effect, offering support to accept H_9_. Table 5 shows the path coefficient results.

### 4.4 Moderating effect

The moderating effect (as presented in Table 6) of online reviews' usefulness on the relationship between the intention to use OHBPs and the usage of OHBPs was evaluated. The result exposed an insignificant moderating effect of UOR on the relationship between IHB and UHB (*β* = –0.039, CI min = –0.100, CI max = 0.024, *p* = 0.161). It provides no support for HM_1_. Similarly, moderating effect of the volume of online reviews on the relationship between the intention to use and usage of OHBPs was also assessed. The result revealed that VOR did not moderate the relationship between IHB and UHB (*β* = 0.020, CI min = –0.049, CI max = 0.074, *p* = 0.304), hence, not supporting the acceptance of HM_2_.

**Table 6:** Moderation analysis.

	Β	CI-min	CI-max	t-value	Sig.	Decision
IHBxUOR → UHB	-0.039	-0.100	0.024	0.993	0.161	No Moderation
IHBxVOR → UHB	0.020	-0.049	0.074	0.514	0.304	No Moderation

*Note:* IHB: Intention to use OHBPs; UOR: Usefulness of online reviews; VOR: Volume of online reviews; UHB: Usage of OHBPs.

### 4.5 Importance-performance analysis

The importance-performance matrix (IPMA) results (Table 7) exposed that the perception of OHBPs' benefit was the essential factor in the performance of the usage of the OHBPs with a score of 78.190, followed by the intention to use the OHBPs with a score of 77.540. The third most important reason for the performance of the usage of OHBPs was the volume of online reviews with a score of 77.540. Finally, the system quality and usefulness of the OHBPs were the fourth and fifth significant factors for the performance of the usage of the OHBPs, respectively.

**Table 7:** Importance-performance matrix.

Factors of Usage	Total effect	Performance
INQ	0.032	71.869
IGH	-0.010	70.260
PRB	0.157	78.199
PRK	-0.025	65.626
SQL	0.340	76.746
SRL	0.121	73.357
IHB	0.496	77.925
UOR	0.110	74.323
VOR	0.127	77.540

*Note:* INQ: Information Quality; IGH: Integrity of online hotel booking; PRB: Perceived benefits; PRK: Perceived risk; SQL: System quality, SRL: Service quality; IHB: Intention to use online hotel booking; UOR: Usefulness of online reviews; VOR: Volume of online reviews; UHB: Usage of OHBPs.

### 4.6 Predictive assessment

The model's predictive power was evaluated with the PLS_Predict_ with 10 folds and 10 repetitions. The assessment evaluated the performance of the PLS model with new predictive observations than the linear model (LM). Only a few endogenous constructs' RMSE for PLS-SEM indicators outperformed the naïve benchmark (Shmueli et al., 2019) for the intention to use OHBPs. Therefore, the Q^2^_predict_ value was above 0. Then, the prediction error was analyzed in detail to evaluate the relevant prediction statistic. Therefore, the study evaluated the predictive power constructed on the RMSE scores (Shmueli et al., 2019). The results showed that the intention to use OHBPs had low predictive power, as most of the LM naïve benchmarks yielded fewer errors than the PLS-SEM. Nevertheless, the usage of OHBPs had high predictive power as most of the PLS-SEM yielded fewer errors than the LM naïve benchmarks. The results are provided in Table 8.

**Table 8:** Predictive model assessment.

	Q^2^_Predict_	RMSE (PLS-SEM)	RMSE (LM)	Difference	Predictive Power
IHB1	0.319	0.997	0.971	0.026	
IHB2	0.311	1.004	0.931	0.073	Low Predictive Power
IHB3	0.331	0.965	0.975	-0.010	
IHB4	0.336	0.930	0.870	0.060	
IHB5	0.352	0.973	0.878	0.095	
UHB	0.269	0.979	0.989	0.021	High Predictive power

*Note:* INQ: Information Quality; IGH: Integrity of online hotel booking; PRB: Perceived benefits; PRK: Perceived risk; SQL: System quality, SRL: Service quality; IHB: Intention to use online hotel booking; UOR: Usefulness of online reviews; VOR: Volume of online reviews; UHB: Usage of OHBPs; MAE: Mean Absolute Error; RMSE: Root Mean Squared Error; PLS-SEM: Partial Least Squares – Structural Equation Modelling; LM: Linear Regression Model.

## 5 Discussion

The current study primarily examined the intention to use and the usage of OHBPs among Malaysian respondents with online reviews from customers about the hotel booking websites. The study’s results demonstrated that perceived benefits, system quality, and service quality significantly influenced the intention to use OHBPs. The findings agreed with the outcomes in Kim et al. (2019) and Park and Huang (2017) that online hotel booking offered the benefits of timesaving, convenience, and readily available information and harnessed the intention to use information online hotel booking.

The present study confirmed that the OHBPs' system and service quality significantly predicted the intention to use the OHBPs. The study results concurred with the findings of Elwalda et al. (2016) and Ozturk et al. (2017) that online platforms' system level and service quality promoted the intention to use the OHBPs. Besides, the ease of use, promptness, and error-free responses greatly facilitated the users and built the necessary inclination to use OHBPs (Agag et al., 2020).

Nonetheless, this study’s results showed that information quality, integrity, and perceived risk of the OHBPs had an insignificant effect on the intention to use OHBPs. This outcome agrees with the verdict of Tran et al. (2017) that online consumers are looking for complete information that helps to perceive the value of the product or service and leads to having a better understanding of the product or services offered. Incomplete information offered by online sellers causes a lack of interest in using online shopping platforms.

Thus, online users are looking for reliable and trustworthy information depicting the value of the product and services offered. The study findings suggest that Malaysian consumers find that the available information on hotel booking sites lacks integrity, leading to a decline in the intention to use the OHBPs. This result matches that of Park and Huang (2017) that the information available on online hotel booking sites lacks integrity and cannot prompt online buyers to take an interest in performing online hotel booking. Furthermore, the study results agreed with the findings of Gurung et al. (2016) that the perception of risk lowered the intention to use OHBPs. Prospective consumers feel uncomfortable and show a lack of trust in the online seller, and this lack of trust and hesitation leads to not using the OHBPs.

Next, the study analysis depicted that the user reviews, the volume of the user reviews, and the intention to use the OHBPs significantly influenced the usage of the OHBPs. This study’s finding matched the result of Tien et al. (2019) that the eWOM on social media and hotel booking platforms significantly influenced hotel booking. In the sharing economy, consumer to consumer information sharing and WOM help select and plan travels (Tran et al., 2017). Online reviews offer rich information about the location, hotel services, hotel location, and other allied services and stimulate travellers to judge and book the hotel online (Zhu et al., 2016).

### 5.1 Theoretical implications

The current work theoretically contributes to using the effective mix of the influential website attributes that harness the intention of using the online hotel booking platforms. The website system and service level attributes are more important in forming the intention to use the OHBPs. Currently, the information quality and integrity of online hotel booking websites are not good and insignificantly impact the intention to use the intention to use the online hotel booking system. On the other hand, the usefulness and volume of online reviews theoretically included testing the moderated role in the formation of use behaviour for OHBPs among tourists.

### 5.2 Policy and managerial implications

The OHBPs and top hotel management need to understand the increasing interest of hotel users in online hotel booking. Online hotel booking offers multiple benefits over conventional hotel booking systems. Nonetheless, hotel management must be very careful with the efficacy of the information regarding hotel services, locations, and pricing and offer comprehensive information about hotel services (Park and Huang, 2017). Currently, Malaysian hotel booking consumers prefer to have more all-inclusive and valid information about the hotels' services. The richness of information helps build prospective values in hotel booking customers' minds. The OHBPs also need to provide security and peace of mind to the online hotel booking customers by offering multiple and secure payment systems and guaranteeing the privacy of the customers' personal information (Agag et al., 2019). Moreover, the OHBPs must uplift the system, service level, and website quality attributes to enhance consumers' confidence in online hotel booking.

Furthermore, OHBPs must offer the rating of the hotel services and rank the hotels based on previous customers' experiences. It provides a correct and realistic outcome of the hotels for the prospective hotel customers. It improves the prospective online hotel booking customers' confidence to book a hotel and have a realistic expectation of the hotel services. Online reviews volume and usefulness still need attention from the hotel management. The top management must offer a discount to encourage the hotel customers to offer reviews and feedback. The reviews and volume can offer better insight into the hotel facilities and services. Also, hotel management can offer 3D panorama views of the hotel and services with customer videos that can help enhance customer-level service expectations and enable the hotels to transform prospective customers visiting the websites into actual customers.

Three limitations were noted in this current study. First, the study concentrated on the intention to use and use OHBPs. This study was designed to show that intention led to the usage of the OHBPs. Nonetheless, the OHBPs' attributes directly influenced the usage of OHBPs. Furthermore, the online reviews' quality and quantity also influenced the intention to use OHBPs. Therefore, future studies need to evaluate the effect of the intention to use and usage of OHBPs on hotel booking websites' attributes and users' online reviews. Secondly, the data were collected from Malaysian respondents; thus, the current work’s generalisation is limited to the Malaysian context only. Future research needs to include samples from neighbouring countries to validate the study model and explore the phenomenon of online hotel booking. Lastly, the current study utilized only a few website attributes and therefore cannot fully expose the process of intention formation to use OHBPs.

## 6 Conclusion

The current work aimed to explore the formation of online hotel booking intention, the effect of user reviews, the volume of online reviews, and the intention and usage of OHBPs. The study’s findings provide an empirical indication that OHBPs need improvement in providing information about the hotel location and prices and provide actual visuals to offer necessary information about the hotel, facilitating online hotel booking. Currently, Malaysian customers are not satisfied with the current OHBPs' integrity, and the perceived risks reveal the insignificant intention to use the OHBPs.

Moreover, the usefulness and volume of the online reviewers by the previous customer are not enough to facilitate the intention into use behaviour for the new customers to use the OHBPs. The hotel management needs to work with the customer and offer better services that can prompt the users to offer timely and quality reviews for the hotel. Keeping a close interaction with the existing and prospective customers may help the hotels to attract and facilitate new customers to use the online hotel booking services.

## Declarations

### Author contribution statement

Anas A. Salameh, Noor Raihani Zainol, Mohd Helmi Ali: Conceived and designed the experiments; Performed the experiments; Wrote the paper.

Abdullah Al Mamun and Naeem Hayat: Conceived and designed the experiments; Analyzed and interpreted the data; Contributed reagents, materials, analysis tools or data; Wrote the paper.

### Funding statement

This research did not receive any specific grant from funding agencies in the public, commercial, or not-for-profit sectors.

### Data availability statement

Data included in article/supp. material/referenced in article.

### Declaration of interest’s statement

The authors declare no conflict of interest.

### Additional information

No additional information is available for this paper.
